# Task offloading and resource allocation for cooperative communication and sensing in edge computing for mine

**DOI:** 10.1038/s41598-026-52020-w

**Published:** 2026-05-15

**Authors:** Wanbo Zheng, Qiuping Yang, Kerong Chen, Siqi Li, Yunni Xia, Kunyin Guo

**Affiliations:** 1 The Faculty of Science, Kunming University of Science and Technology, Kunming, 650500 China; 2 Yunnan Key Laboratory of Complex Systems and Brain-Inspired Intelligence, Kunming, China; 3 The School of Computer Science, Chongqing University, Chongqing, 400044 China

**Keywords:** Energy science and technology, Engineering, Mathematics and computing

## Abstract

With the growing demand for computation-intensive and latency-critical tasks in intelligent mining, the computing capabilities of terminal devices are becoming increasingly inadequate. Consequently, task offloading has emerged as a vital mechanism. However, existing approaches often depend on centralized resource allocation algorithms, which tend to produce suboptimal assignment decisions. As a result, tasks are frequently offloaded to inappropriate edge servers that become unstable under heavy upload traffic, leading to higher latency and increased energy consumption. To effectively assess system performance, we introduce the Overall Utility Value (OUV), which balances system delay and energy usage. In this paper, we present an edge computing task-offloading framework tailored for mining scenarios, which comprises a central control unit, distributed service nodes, and a large number of terminal devices. By leveraging the environmental awareness of the service nodes, we present a Cooperative Communication and Sensing Task Offloading Scheme (CCTS) designed to minimize both system latency (SL) and system energy consumption (SEC) through optimized task allocation and wireless bandwidth ratios. To tackle this optimization problem, we develop an Improved Gray Wolf Optimization algorithm integrated with a Feasibility Checking Algorithm (IGWO-FCA). Simulation results demonstrate that the IGWO-FCA achieves the lowest OUV, validating its effectiveness.

## Introduction

In recent years, the continuous advancement of global intelligence has driven the widespread adoption of smart devices and applications across various sectors. By leveraging information and communication technology, these innovations have significantly accelerated the growth of the digital sector and supported industrial production^1^. To meet the needs for task timing and resources in delay-sensitive, compute-intensive Industrial Internet of Things (IIoT) tasks, the computing model has evolved from cloud computing to edge computing, which is deployed much closer to user devices^2,3^. Smart mining mainly relies on IIoT, edge computing, big data, and modern mining equipment to realize real-time monitoring, smarter analysis, quicker processing, and better decisions in production, safety supervision, maintenance, and logistics. In this setup, edge computing is particularly important: it pushes computation close to the data source so that urgent tasks can still be handled instantly even when the underground network becomes unreliable^4^. To meet the complex task requirements for latency and energy consumption in mining networks, tasks can be offloaded to Mobile Edge Computing (MEC) servers and service terminal (ST) for collaborative execution^5^.

In the traditional task offloading model, tasks are offloaded to the MEC server. However, when the computational load is too high, the MEC server often becomes overloaded. In such cases, tasks can be offloaded to nearby terminal devices that have excess computational capacity^6^.Task offloading between the MEC server and terminal devices requires splitting the task into multiple independent parts and distributing them across different computing devices for execution^7^. Terminal devices collaborate with MEC servers for task offloading through the communication link between the task terminal (TaT) and the ST. In addition to executing tasks locally, the TaT can offload tasks to either the ST or the MEC server for further processing^8,9^

Traditional research on task offloading with MEC servers has made significant progress, particularly in binary studies using the 0-1 mode. However, when partial offloading is involved, especially with multiple optimization metrics and continuous variables, the problem becomes much more complex. A large body of research has focused on minimizing both energy consumption and latency in these scenarios. In^10^, a meta-heuristic Edge Genetic Algorithm (EdgeGA) was proposed, designed to tackle computational resource challenges in vehicles by focusing on the time complexity of Binary Integer Programming (BIP). Furthermore, containerized orchestration mechanisms running on Kubernetes have been introduced to address task offloading issues. In^11^, a heuristic algorithm was proposed to reduce system costs and latency in a Non-Orthogonal Multiple Access (NOMA)-based Vehicular Edge Computing (VEC) model by optimizing offloading decisions and resource allocation. In^12^, the choice between local processing and cloud offloading has been formulated as an optimization problem and solved exactly via enumeration or branch-and-bound approaches that account for latency and energy constraints.

Many studies have explored scenarios where ST have excess computing resources. In task offloading scenarios, ST can also serve as offloading targets. By tapping into the idle computing resources of ST, the computational burden on MEC servers can be eased. In^13^, the problem of minimizing weighted sum energy in a multi-user MEC network under latency constraints was studied, and a joint optimization framework was proposed, taking into account both computational and communication resources for each user in the network. In^14^ and^15^, a heuristic algorithm was proposed for the joint allocation of computational and communication resources in a single-user MEC network, with the goal of minimizing task latency. In^16^, a resource allocation scheme for energy harvesting was proposed to improve energy efficiency in digital twin IIoT systems. In^17^, a multi-objective optimization framework was proposed for task offloading and caching in device-to-device communication within authorized MEC networks.

In addition to exploring collaborative task offloading between MEC servers and ST, task partitioning and offloading have also become a recent research focus. In this approach, tasks are divided into independent subtasks, which are then assigned to the TaT, ST, or MEC servers for processing. This partitioning and collaborative model effectively enables the completion of computational tasks. In^18^, the spectrum and energy efficiency of non-orthogonal next-generation multiuser uplink wireless networks were jointly optimized. In^19^, a game-theoretic approach to task offloading is presented, explicitly taking into account both millimeter-wave and cellular modes within 5G New Radio Vehicle-to-Everything (NR-V2X) wireless access networks. In^20^, a Quantum Reinforcement Learning (QRL) scheme for task offloading is proposed. First, the resource management scheme is framed as a delay optimization problem, which is then converted into a Markov Decision Process (MDP). Next, QRL is used to determine the optimal resource allocation strategy. In^21^, Considering the bandwidth resource allocation strategy, an online fronthaul scheduling approach was proposed to manage link status and requests, ultimately maximizing average profit.

## Related work

### Task offloading in MEC systems

Task offloading has been widely studied as an effective solution to reduce latency and energy consumption in MEC servers. Early studies mainly focused on binary offloading strategies, where tasks are executed either locally or entirely offloaded to edge servers. Subsequent research introduced partial offloading mechanisms that allow tasks to be divided into multiple components for flexible execution across local devices and MEC servers^10–12^. These approaches improve system performance by balancing computation workload and communication cost. However, most existing works focus primarily on single-device offloading decisions and do not fully exploit collaborative opportunities among multiple devices in distributed environments.

### Collaborative and partial offloading

To further enhance system efficiency, collaborative task offloading among multiple devices has attracted increasing attention. Some studies investigate cooperative computing frameworks in which neighboring devices assist in executing subtasks^13–17^. Other works assume that tasks can be arbitrarily partitioned into independent subtasks that can be processed in parallel across different computing nodes^24,25^. These studies demonstrate that collaborative offloading can effectively reduce computation latency and energy consumption. Nevertheless, most existing collaborative offloading models primarily optimize task partition ratios or transmission strategies, while the potential of integrating sensing information into the offloading process has not been sufficiently explored.

### Learning-based offloading approaches

Recently, learning-based approaches have been proposed to address the dynamic and uncertain nature of MEC environments. In particular, deep reinforcement learning (DRL) techniques such as deep Q-networks (DQN) and proximal policy optimization (PPO) have been applied to optimize task offloading and resource allocation decisions^22,23^. These methods can adapt to time-varying network conditions by learning policies from historical interactions. However, DRL-based solutions often require extensive training data, careful reward design, and significant computational resources, which may limit their practicality in real-time industrial scenarios such as smart mining.

### Research gap and our positioning

Different from the above studies, this paper proposes a CCTS for MEC-enabled smart mining environments. The proposed framework introduces a sensing-assisted transmission mechanism that allows task terminals to select between direct data transmission and collaborative sensing instruction transmission. By jointly optimizing task partitioning, transmission mode selection, and bandwidth allocation, the proposed approach effectively exploits both communication and sensing resources in the system. IGWO-FCA is further developed to efficiently solve the resulting optimization problem.

## Motivation and contribution

This paper investigates task offloading and resource allocation in MEC-enabled smart mining environments, where delay-sensitive detection tasks and limited computing resources make efficient offloading essential. Existing approaches primarily address either task partitioning or resource allocation, but seldom leverage the sensing capabilities of service terminals to assist transmission and computation decisions. To address this gap, we propose a CCTS, which adaptively selects the transmission mode for input-data uploads and computation instructions, and jointly optimizes task and bandwidth allocation ratios to minimize the OUV. The MEC server is assumed to be colocated with the Central Control Unit (CU). Terminals and base stations (BS) continuously collect real-time environmental data through sensing devices, which are reported to the CU at regular intervals. This enables the centralized control system to access key information, including wireless transmission capacity, computing load, and computing power. When a TaT has a task to offload, the CU makes the offloading decision based on the overall system state and communicates it to the TaT. During data uploads to service nodes (SN), TaTs can dynamically choose between two transmission methods: uploading input data or transmitting computing instructions. In the first method, the SN directly receives input data, while in the second, the SN uses sensed environmental data as input. Overall, SNs exhibit significant variations in input data volume, transmission capacity, and coordinate transformation rates. Some SNs favor uploading input data, while others prefer transmitting instructions along with sensed data. Accordingly, the transmission strategy for each SN can be adaptively selected, taking into account the objective function, network traffic, and transmission capacity. Most existing MEC offloading schemes focus on either task partitioning or resource allocation, without exploiting sensing-assisted transmission to jointly optimize delay and energy. To overcome this limitation, we propose the CCTS mechanism, which adaptively selects transmission strategies and jointly optimizes task and bandwidth allocation to minimize system OUV. The core contributions of this paper are summarized as follows:

- We design a CCTS that leverages the sensing capabilities of SNs to minimize system OUV. Within the constraints of task execution delay and bandwidth resources, TaTs can dynamically choose between uploading input data or transmitting computing instructions. Fundamentally distinguishing our approach from existing reactive offloading models, this sensing-assisted transmission choice proactively utilizes environmental context (e.g., utilizing sensed data for coordinate transformations in CSIT) as a substitute for heavy data offloading, rather than relying solely on instantaneous communication metrics.
- We introduce an IGWO-FCA algorithm to solve the optimization problem. First, Improved Gray Wolf Optimization generates a candidate population of offloading decisions, followed by a feasibility check to ensure all constraints are satisfied. By analyzing the relationship among task ratio, bandwidth ratio, and OUV, the problem is reformulated over the bandwidth ratio and solved using the Gradient Projection Method (GPM).
- Simulation results demonstrate that CCTS achieves lower OUV than benchmark schemes. The IGWO-FCA algorithm outperforms multiple baselines, including local computation, partial noncooperative offloading, and binary offloading.

The remainder of this paper is organized as follows. Section 4 introduces the system and network model for mine-edge computing within IIoT, where the CCTS is formulated. Section 5 presents the IGWO-FCA algorithm. Section 6 validates the performance of both CCTS and IGWO-FCA. Finally, Section 7 concludes the paper and discusses potential directions for future research.

**Fig. 1:**
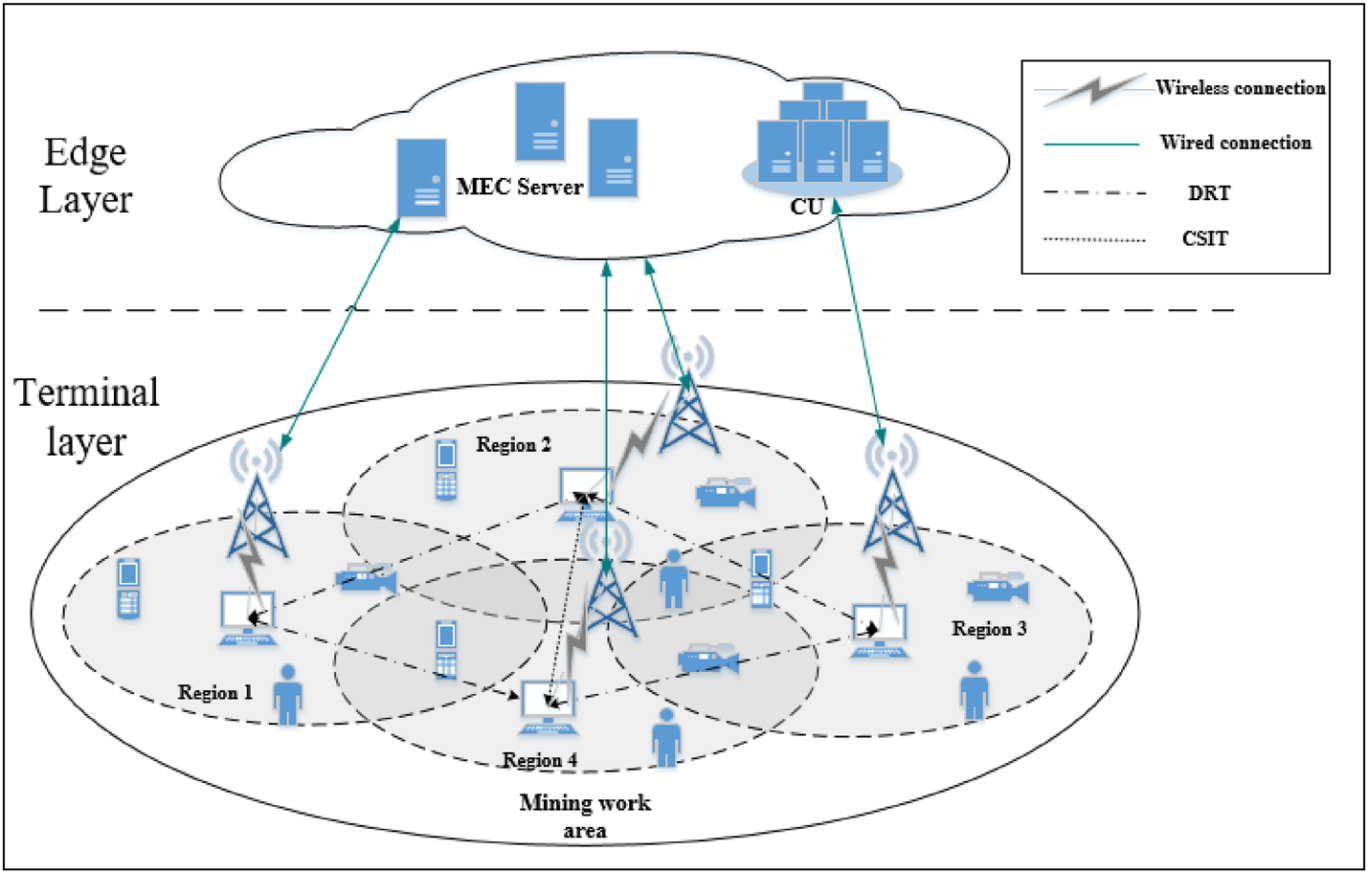
The architecture of mine edge computing.

## System and network model

The smart mine edge computing framework is illustrated in Fig. 1 It includes a CU and several Service BS that are deployed across different work areas within the mine. The BS have a certain storage capacity and can store data collected by User Equipment (UE) in their respective work areas. For data transmission, the CU and BS are connected by wires. The CU serves as the information control and management center, collocated with the MEC server. The TaT uses Frequency Division Multiple Access (FDMA) technology to communicate with both the Service Link and the BS simultaneously. Moreover, the uplink and feedback processes employ a Time Division Duplex (TDD) mode.

For tasks offloaded by UEs within the coverage area of a BS, the tasks are characterized by their intensity and latency sensitivity during the transmission of terminal data and the task offloading process. For instance, in emergency scenarios, the quick and accurate location of personnel needs to be processed promptly and effectively.

In fact, to ensure safety during the operation of smart mines, enough sensor devices have been deployed in them, leading to a large volume of data collection and complex task types. In addition, BSs are typically deployed in a distributed manner across the mining work areas, sensing environmental information within their coverage areas and connected to MEC servers via wired links. For the TaT, the environmental information obtained by BSs and SNs in a mining work area is similar due to their sensing capabilities.To ensure consistency between the environmental data sensed by the ST and MEC servers and that perceived by the TaT, the sensed data must undergo preprocessing through coordinate transformation. This can be accomplished by performing atrix operations using the terminal coordinates provided in the computing instructions.^26^

Denote the task terminal as $D_0$, the service terminal as $D_n$, where $n \in N$ and {N = 1, 2, ..., N } and the MEC server as $D_{N+1}$. To minimize the overall OUV of the entire task, it is necessary to jointly optimize the transmission strategy and task offloading. $a_n \in [0,1]$,{n = 0, 1, ..., N+1 } and bandwidth allocation ratio $b_n \in [0,1]$,{n = 0, 1, ..., N+1} for each node. Additionally, we use $\textrm{Task} = (S_{\textrm{data}}, S_{\textrm{instr}}, M)$ to describe the task, where $S_{data}$ represents the size of input data, $S_{instr}$ represents the size of computational instructions in terms of cycles per bit, and *M* represents the computational intensity of the task. The parameter M primarily depends on the nature of the task itself.^27^

The task of offloading decisions is determined by $\{a_n\}_{n=0}^{N+1}$ and $\{b_n\}_{n=0}^{N+1}$ the choice of transmission strategy. A low-complexity optimization algorithm can lower the time for offloading decisions and the energy required for executing offloaded tasks. At the same time, parallel execution and high-performance servers help to further cut decision latency and energy usage. As a result, the objective is simplified to minimizing the OUV, which includes the time taken to upload input data and instructions, compute subtasks, and provide output feedback.

## Problem formulation

### CCTS mechanism

During the process of offloading tasks from the TaT to the ST or MEC server, an adaptive selection is made between two transmission modes. The first is Direct Regular Transmission (DRT): when the task is offloaded to the ST, the TaT directly transmits the input data to the ST via a wired link. When the task is offloaded to the MEC servers, the ST sends the input data to the BS through a wireless link, which then forwards the data to the MEC server via a wired connection. The second mode is Collaborative Sensing Instruction Transmission (CSIT): in this mode, the ST receives computing instructions from the TaT via a wireless link and adjusts its sensed environmental data to match the coordinate system and offloaded subtask, enabling it to perform the task. If the MEC server is selected, instruction transmission is not required; instead, the BS simply uploads its sensed environmental data to the MEC server via a wired link. The reason is that, once the CU has reached an offloading decision, it is immediately relayed to the TaT through the BS. While caching the offloading strategy, the BS can sense and preprocess the relevant environmental data and execute the subtask.

**Fig. 2:**
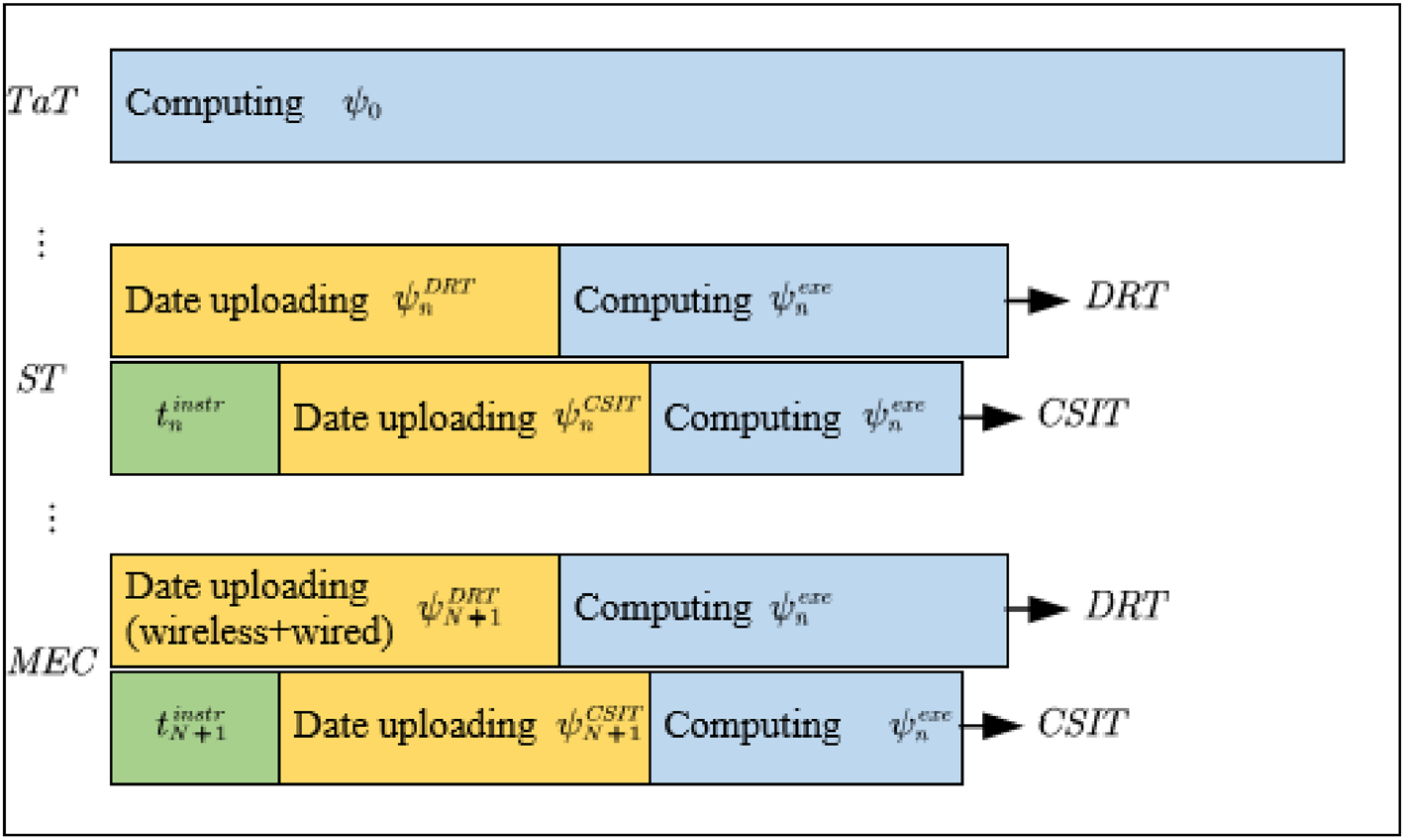
CCTS Mechanism.

To meet the dynamic requirements in the task of offloading the transmission process, we propose a CCTS. During the task offloading process, this mechanism adaptively selects the transmission method for each TaT based on the task transmission requirements, either DRT or CSIT, to minimize the system’s OUV. Unlike traditional transmission mechanisms, CCTS leverages the sensing capabilities of SNs to determine whether to directly upload data or transmit computing instructions for task offloading. While prior collaborative works primarily optimize partition ratios based on channel states, our sensing-assisted mechanism intricately couples sensing and transmission. Specifically, the availability and processing complexity of sensed environmental data directly dictate the optimal transmission mode (DRT or CSIT), enabling the system to preemptively overcome wireless bandwidth bottlenecks in complex mining environments. The CCTS mechanism is shown in Fig. 2. The following steps, 1 to 5, specifically describe the task of the offloading process of CCTS:

Step 1: The TaT formulates an offloading task and reports its characteristics to the CU, including the sizes of the input and output data, the required number of computation cycles, and other pertinent parameters.

Step 2: The CU decides how to offload tasks by considering the task information, as well as the current status of the TaT and the SNs in the system. More specifically, it determines how to divide the task workload and how to allocate the available bandwidth; at the same time, it selects an appropriate transmission strategy.

Step 3: The offloading decision is stored in both the CU and BS, and then transmitted to the TaT through the CU.

Step 4: The TaT splits the task according to the task allocation ratio from the offloading decision and assigns the subtasks to either local execution or execution by the SNs.

Step 5: When DRT is selected for data transmission, the SNs directly receive the input data from the TaT and immediately execute the computing task. When CSIT is selected for data transmission, the SNs perform a coordinate transformation on the sensed environmental data based on the coordinate information contained in the computing instructions and then begin computation.

The following sections will analyze the response time and energy consumption of parallel subtasks across the task terminal, service terminal, and MEC server within an intelligent mining environment.

### The OUV of the task terminal

When the terminal device $D_0$ a executes a subtask, the OUV is solely dependent on the computational capability $f_0$ of the terminal device and the allocated task data volume $a_0$ $S_{data}$. There is variability in the computational capabilities of different devices. The time taken by terminal devices to execute subtasks can be expressed as follows:

1 $$\begin{aligned} t_0^{\text {exe}}(a_0) = \frac{a_0 S_{\text {data}} M}{f_0} \end{aligned}$$

The energy consumed by the terminal device to execute a subtask is given by:

2 $$\begin{aligned} e_0^{\text {exe}}(a_0) = k_0 f_0^2 a_0 S_{\text {data}} M \end{aligned}$$

where *k* is determined by the chip architecture of the device. Using the above formula, we can derive the OUV for executing the subtask at the terminal device as follows:

3 $$\begin{aligned} \psi _0(a_0) = \mu t_0^{\text {exe}}(a_0) + v e_0^{\text {exe}}(a_0) \end{aligned}$$

Here, the time and energy consumption for local subtask execution are weighted by the coefficients $\mu$ and $\nu$, respectively. The relationship between these two weighting factors is typically defined as follows:

4 $$\begin{aligned} \mu + v = 1, \quad 0 \le \mu , v \le 1 \end{aligned}$$

Observation reveals that $\mu$ and $\nu$ can adjust the total consumption of the computational task. A larger $\mu$ implies greater emphasis on latency, which is typically configured for latency-critical mining tasks such as emergency disaster alerts or real-time personnel positioning. Conversely, a larger $\nu$ implies a greater focus on energy consumption, which is suitable for routine environmental monitoring tasks to prolong the battery life of distributed terminals.

### The OUV of the service terminal

When a subtask is executed on the service terminal $D_n$, the input data or computational directives need to be delivered to the terminal over a wireless connection. Following this, $D_n$ leverages the received input data or sensor readings derived from coordinate conversions to carry out the subtask. According to much of the existing literature, when the amount of data returned is small, the energy consumption and delay of sending back the results are usually treated as negligible. Following this common assumption, we also consider the size of all computation outputs in this paper to be very small; therefore, we ignore both the transmission delay and the power consumption associated with returning the results.

- Uploading Model: Firstly, the Wireless upload rate $R_{D_0 \rightarrow D_n}$ from terminal $D_0$ to the service terminal $D_n$, is defined as follows:
  5 $$\begin{aligned} R_{D_0 \rightarrow D_n} = b_n B_{\text {total}} \log _2 \left( 1 + \frac{p_n h_n}{\sigma _n^2} \right) \end{aligned}$$

Where $b_n$ denotes the fraction of the overall wireless bandwidth assigned to $D_n$ , $p_n$ represents the transmission power, $\sigma _n^2$ indicates the noise variance, $h_n$ signifies the channel coefficient linking $D_0$ and $D_n$, $B_{total}$ stands for the system’s aggregate wireless bandwidth capacity. When the transmission strategy is DRT, the transmission time from $D_0$ to $D_n$ is:

6 $$\begin{aligned} t_n^{\text {DRT}}(a_n, b_n) = \frac{a_n S_{\text {data}}}{R_{D_0 \rightarrow D_n}} \end{aligned}$$

The resulting transmission energy consumption is:

7 $$\begin{aligned} e_n^{\text {DRT}}(a_n, b_n) = p_n t_n^{\text {DRT}}(a_n, b_n) \end{aligned}$$

Based on the above formula, we can derive the OUV of the subtask upload process under the DRT scenario as follows:

8 $$\begin{aligned} \psi _n^{\text {DRT}}(a_n, b_n) = \mu t_n^{\text {DRT}}(a_n, b_n) + v e_n^{\text {DRT}}(a_n, b_n) \end{aligned}$$

Similarly to the DRT case, when the transmission strategy is CSIT, $D_n$ performs coordinate transformation on perceived environmental data upon receiving the computing instructions. The transmission process involves two key components: the transmission of computing instructions and the coordinate transformation of the data. For the CSIT scenario, the transmission time to $D_n$ is:

9 $$\begin{aligned} t_n^{\text {CSIT}}(a_n, b_n) = \frac{S_{\text {instr}}}{R_{D_0 \rightarrow D_n}} + \frac{a_n S_{\text {data}} M_{\text {tra}}}{f_n} \end{aligned}$$

In the equation, $S_{instr}$ represents several coordinates containing environmental information, which typically range from hundreds to thousands of bits; $M_{tra}$ denotes the computational complexity associated with the coordinate conversion, and $f_n$ represents the CPU cycles of $D_n$. The resulting transmission energy consumption is:

10 $$\begin{aligned} e_n^{\text {CSIT}}(a_n, b_n) = \frac{p_n S_{\text {instr}}}{R_{D_0 \rightarrow D_n}} + k_n(f_n)^2 a_n S_{\text {data}} M_{\text {tra}} \end{aligned}$$

Based on the above formula, we can derive the OUV of the subtask upload process under the CSIT scenario as follows:

11 $$\begin{aligned} \psi _n^{\text {CSIT}}(a_n, b_n) = \mu t_n^{\text {CSIT}}(a_n, b_n) + v e_n^{\text {CSIT}}(a_n, b_n) \end{aligned}$$

The proposed CCTS selects the one with the lower OUV for task data transmission by comparing the OUVs of the DRT and CSIT mechanisms. Therefore, the OUV of the upload process from $D_0$ to $D_n$ can be succinctly expressed as:

12 $$\begin{aligned} \psi _n^{\text {trans}}(a_n, b_n) = \min \{\psi _n^{\text {DRT}}(a_n, b_n), \psi _n^{\text {CSIT}}(a_n, b_n)\} \end{aligned}$$

Therefore, the CSIT mode is selected only when the reduction in wireless transmission cost is sufficient to compensate for the additional coordinate transformation delay and energy consumption; otherwise, the DRT mode is preferred.

- Computing Model: After the upload is complete, $D_0$ it executes the assigned subtasks in parallel and performs the computation. The following equation gives the computation time:
  13 $$\begin{aligned} t_n^{\text {exe}}(a_n) = \frac{a_n S_{\text {data}} M}{f_n} \end{aligned}$$

Computational energy consumption is:

14 $$\begin{aligned} e_n^{\text {exe}}(a_n) = k_n f_n^2 a_n S_{\text {data}} M \end{aligned}$$

The OUV is calculated as follows:

15 $$\begin{aligned} \psi _n^{\text {exe}}(a_n) = \mu t_n^{\text {exe}}(a_n) + v e_n^{\text {exe}}(a_n) \end{aligned}$$

In summary, the execution process of the subtask at the service terminal includes both the upload and computation processes. Thus, the OUV for the service terminal to complete the subtask is:

16 $$\begin{aligned} \psi _n(a_n, b_n) = \psi _n^{\text {trans}}(a_n, b_n) + \psi _n^{\text {exe}}(a_n) \end{aligned}$$

### The OUV of the MEC server

When the subtask is executed on the MEC server, it is essential to analyze the wireless and wired links separately. The MEC server is connected to the BS via a wired link, while the BS communicates with the task terminal through a wireless link. Therefore, when discussing the subtask execution process, it is essential to consider both types of links.

- Uploading Model: Assuming the bandwidth allocation ratio from the task terminal to the BS is $b_{N+1}$, with noise power $\sigma _{N+1}$ and uplink transmission power $p_{N+1}$, and the channel gain from the task terminal to the BS is $h_{N+1}$, the upload rate from the task terminal to the BS can be expressed as follows:
  17 $$\begin{aligned} R_{D_0 \rightarrow D_{N+1}} = b_{N+1} B_{\text {total}} \log _2 \left( 1 + \frac{p_{N+1} h_{N+1}}{\sigma _N^2} \right) \end{aligned}$$
  First, for the DRT transmission strategy, the time required to upload the subtask to the MEC server is expressed by the following equation:
  18 $$\begin{aligned} t_{N+1}^{\text {DRT}}(a_{N+1}, b_{N+1}) = \frac{a_{N+1} S_{\text {data}}}{R_{D_0 \rightarrow D_{N+1}}} + \frac{a_{N+1} S_{\text {data}}}{R_{\text {wired}}} \end{aligned}$$
  In the equation, $a_{N+1}$ $S_{data}$ represents the amount of subtask data assigned to the MEC server, $R_{wired}$ denotes the wired transmission rate between the MEC server and the BS, and $p_{N+1}$ represents the uplink transmission power of the device. In 18, the first term represents the wireless transmission time from the task terminal to the MEC server, while the second term represents the transmission time through the wired link between the MEC server and the BS. The following equation calculates the upload energy consumption in the case of DRT:
  19 $$\begin{aligned} e_{N+1}^{\text {DRT}}(a_{N+1}, b_{N+1}) = \frac{p_{N+1}a_{N+1}S_{\text {data}}}{R_{D_0 \rightarrow D_{N+1}}} + \frac{p_{\text {wired}}a_{N+1}S_{\text {data}}}{R_{\text {wired}}} \end{aligned}$$
  Based on the above formula, we can derive the OUV of the subtask upload process under the DRT scenario as follows:
  20 $$\begin{aligned} \psi _{N+1}^{\text {DRT}}(a_{N+1}, b_{N+1}) = \mu t_{N+1}^{\text {DRT}}(a_{N+1}, b_{N+1}) + v e_{N+1}^{\text {DRT}}(a_{N+1}, b_{N+1}) \end{aligned}$$
  Next, we analyze the upload time under the CSIT strategy. Because the offloading policy determined by the CU is already cached at both the MEC server and the BS, once the task terminal uploads the computing instructions to the BS, the BS only needs to forward the sensed environmental data to the MEC server over the wired link. The MEC server then performs the coordinate transformation. The upload time is given by the following equation:
  21 $$\begin{aligned} t_{N+1}^{\text {CSIT}}(a_{N+1}, b_{N+1}) = \frac{S_{\text {instr}}}{R_{D_0 \rightarrow D_{N+1}}} + \frac{a_{N+1}S_{\text {data}}}{R_{\text {wired}}} + \frac{a_{N+1}S_{\text {data}}M_{\text {tra}}}{f_{N+1}} \end{aligned}$$
  The energy consumption for uploading is:
  22 $$\begin{aligned} e_{N+1}^{\text {CSIT}}(a_{N+1}, b_{N+1}) = \frac{p_{N+1}S_{\text {instr}}}{R_{D_0 \rightarrow D_{N+1}}} + \frac{p_{\text {wired}}a_{N+1}S_{\text {data}}}{R_{\text {wired}}} + k_{N+1}(f_{N+1})^2 a_{N+1}S_{\text {data}}M_{\text {tra}} \end{aligned}$$
  The OUV of uploading under the CSIT strategy is given by:
  23 $$\begin{aligned} \psi _{N+1}^{\text {CSIT}}(a_{N+1}) = \mu t_{N+1}^{\text {CSIT}}(a_{N+1}) + v e_{N+1}^{\text {CSIT}}(a_{N+1}) \end{aligned}$$
  For the transmission strategy at the MEC server, we continue to select the upload mechanism with the lower OUV. Thus, the OUV of data upload for the subtask at the MEC server can be expressed as:
  24 $$\begin{aligned} \psi _{N+1}^{\text {trans}}(a_{N+1}, b_{N+1}) = \min \{\psi _{N+1}^{\text {DRT}}(a_{N+1}, b_{N+1}), \psi _{N+1}^{\text {CSIT}}(a_{N+1})\} \end{aligned}$$
- Computing Model: With the input data provided, the execution duration on the MEC server for processing its designated subtasks is:
  25 $$\begin{aligned} t_{N+1}^{\text {exe}}(a_{N+1}) = \frac{a_{N+1}S_{\text {data}}M}{f_{N+1}} \end{aligned}$$
  The energy consumption for computation is given by:
  26 $$\begin{aligned} e_{N+1}^{\text {exe}}(a_{N+1}) = k_{N+1}(f_{N+1})^2 a_{N+1}S_{\text {data}}M \end{aligned}$$
  The OUV is calculated as follows:
  27 $$\begin{aligned} \psi _{N+1}^{\text {exe}}(a_{N+1}) = \mu t_{N+1}^{\text {exe}}(a_{N+1}) + v e_{N+1}^{\text {exe}}(a_{N+1}) \end{aligned}$$
  In summary, executing a subtask on the MEC server comprises both the upload and computation phases. Accordingly, the OUV for MEC-side completion of the subtask is:
  28 $$\begin{aligned} \psi _{N+1}(a_{N+1}, b_{N+1}) = \psi _{N+1}^{\text {trans}}(a_{N+1}, b_{N+1}) + \psi _{N+1}^{\text {exe}}(a_{N+1}) \end{aligned}$$

### Problem formulation

This study aims to reduce the system OUV through the joint design of offloading decisions, the task allocation ratio $a_n$, and the bandwidth assignment ratio $b_n$. The corresponding optimization model is given by:

P1 $$\begin{aligned} \begin{aligned} \underset{\boldsymbol{a},\boldsymbol{b}}{\min }\psi \left( \boldsymbol{a},\boldsymbol{b} \right) =\min \sum _{n=0}^{N+1}{\psi _n\left( a_n,b_n \right) }\\ C1:\sum _{n=0}^{N+1}{a_n=1}\\ C2:0\leqslant \sum _{n=0}^{N+1}{b_n\leqslant 1}\\ C3:0\leqslant a_n\leqslant 1,n=0,1,\dots ,N+1\\ C4:0\leqslant b_n\leqslant 1,n=1,2,\dots ,N+1 \end{aligned} \end{aligned}$$

Constraint *C*1 specifies that, after task partitioning, the allocated portions must be fully exhausted, meaning that the task-allocation ratios across all nodes collectively equal 1. Constraint *C*2 sets an upper bound on wireless bandwidth allocation, stipulating that the combined bandwidth share over all networks does not exceed 1. Constraint *C*3 ensures that the task allocation ratios are non-negative and do not exceed 1. Constraint *C*4 ensures that the wireless bandwidth allocation ratios are also non-negative and do not exceed 1.

## Problem solution

The decision variables comprise the offloading decisions, the task allocation ratios $a_n$, and the bandwidth allocation ratios $b_n$, The offloading decisions are discrete, whereas, $a_n$ and $b_n$ are continuous. Problem P1 is a mix of discrete and continuous action spaces. Additionally, this problem has a (hidden) combinatorial nature, as each node must choose between uploading data or instructions. Therefore, solving Problem P1 using traditional methods is challenging. In this section, by analyzing Problem P1, we identify a structural property that enables an efficient solution approach. First, we employ an IGWO algorithm to generate a feasible population of offloading decisions. Next, a feasibility check is performed on the allocation relationships within this feasible offloading population to ensure that the constraints are satisfied and that the subtask is completed within the specified threshold. Observing the structural relationship among $a_n$, $b_n$, and $\psi (a,b)$, the feasibility assessment may instead be posed as an optimization problem involving only $b_n$, thus improving the efficiency of thet solution.

### Problem transformation

For each $n = 1, 2, \dots , N+1$ , we analyzed how $a_n$ and $b_n$ are related through $\psi _n (a_n, b_n)$in order to simplify the expression of their relationship.

As seen from 3, the $\psi _0 (a_0, b_0)$ of TaT depends on the task allocation ratio $a_n$. The $\psi _n (a_n, b_n)$ of the ST is given by the following formula:

29 $$\begin{aligned} \psi _n(a_n, b_n) = \min \left\{ c_{n,1} \frac{a_n}{b_n}, \frac{c_{n,2}}{b_n} + c_{n,3} a_n \right\} + c_{n,4} a_n \end{aligned}$$

Where

$$\begin{aligned} c_{n,1}&= \frac{\mu S_{\text {data}}}{B_{\text {total}} \log _2 \left( 1 + \frac{p_n h_n}{\sigma _n^2} \right) } + \frac{\nu p_n S_{\text {data}}}{B_{\text {total}} \log _2 \left( 1 + \frac{p_n h_n}{\sigma _n^2} \right) } \\ c_{n,2}&= \frac{\mu S_{\text {instr}} + \nu p_n S_{\text {instr}}}{b_n B_{\text {total}} \log _2 \left( 1 + \frac{p_n}{h_n \sigma _n^2} \right) } \\ c_{n,3}&= \frac{\mu S_{\text {data}} M_{\text {tra}}}{f_n} + \nu k_n (f_n)^2 S_{\text {data}} M_{\text {tra}} \\ c_{n,4}&= \frac{\mu S_{\text {data}} M}{f_n} + \nu k_n (f_n)^2 S_{\text {data}} M \end{aligned}$$

are all constans.

For the MEC server, $\psi _{N+1} (a_{N+1}, b_{N+1})$ is given by the following equation

30 $$\begin{aligned} \psi _{N+1}(a_{N+1}, b_{N+1}) = \min \left\{ c_{N+1,1} \frac{a_{N+1}}{b_{N+1}} + c_{N+1,2} a_{N+1}, \frac{c_{N+1,3}}{b_{N+1}} + \frac{c_{N+1,3}}{b_{N+1}} + c_{N+1,4} a_{N+1} \right\} + c_{N+1,5} a_{N+1} \end{aligned}$$

Where

$$\begin{aligned} c_{N+1,1}&= \frac{\mu a_{N+1} S_{\text {data}} + \nu p_{N+1} S_{\text {data}}}{B_{\text {total}} \log _2 \left( 1 + \frac{p_{N+1} h_{N+1}}{\sigma ^2_{N+1}} \right) }, \\ c_{N+1,2}&= \frac{\mu S_{\text {data}} + p_{\text {wired}} S_{\text {data}}}{R_{\text {wired}}}, \\ c_{N+1,3}&= \frac{\mu S_{\text {instr}} + \nu p_{N+1} S_{\text {instr}}}{B_{\text {total}} \log _2 \left( 1 + \frac{p_{N+1} h_{N+1}}{\sigma ^2_{N+1}} \right) }, \\ c_{N+1,4}&= \frac{\mu S_{\text {data}} + \nu p_{\text {wired}} S_{\text {data}}}{R_{\text {wired}}} + \frac{\mu S_{\text {data}} M_{\text {tra}}}{f_{N+1}} + \nu k_{N+1} (f_{N+1})^2 S_{\text {data}} M_{\text {tra}}, \\ c_{N+1,5}&= \frac{\mu S_{\text {data}} M}{f_{N+1}} + \nu k_{N+1} (f_{N+1})^2 S_{\text {data}} M_{\text {tra}} \end{aligned}$$

From 29 and 30, it can be seen that the task and bandwidth allocation ratios determine the $\psi _n (a_n, b_n)$ of each node. We will design an algorithm to take advantage of this structural relationship. In this structure, given $a_n$ and $b_n$, the $\psi _n (a_n, b_n)$ of the subtask can be calculated. By comparing DRT and CSIT, the transmission strategy with the smallest $\psi _n (a_n, b_n)$ is selected, thus determining the $\psi _n (a_n, b_n)$ of the node. From the structural relationship, we can observe that $\psi _n (a_n, b_n)$ is a monotonically increasing function of $a_n$, $\psi _n (a_n, b_n)$ while is a monotonically decreasing function of $b_n$.

By analyzing the relationship between $a_n$ and $b_n$, an equivalent optimization problem is derived to ensure that the node can complete the subtask within the given utility value $\psi > 0$. From the relationship between $a_n$ and $b_n$ in equation 3, the unique solution to the equation $\psi _0 (a_0) = \psi$ is given by:

31 $$\begin{aligned} a_0 (\psi ) = \frac{\psi f_0}{\mu S_{\text {data}} M + \nu k_0 f_0^3 S_{\text {data}} M} \end{aligned}$$

Furthermore, given $\psi > 0$ and $b_n$ , since according to 29 and 30, $\psi _n (a_n, b_n)$ is a linear function of $a_n$ , The unique solution for $\psi _n (a_n, b_n) = \psi$ is given by the following equation:

32 $$\begin{aligned} \max \{a_n^{\text {DRT}} (b_n, \psi ), a_n^{\text {CSIT}} (b_n, \psi )\} \end{aligned}$$

Where

$$\begin{aligned} a_n^{\text {DRT}} (b_n, \psi )&= \frac{\psi }{\frac{c_{n,1}}{b_n} + c_{n,4}}, \quad a_n^{\text {CSIT}} (b_n, \psi ) = \frac{\psi - \frac{c_{n,2}}{b_n}}{c_{n,3} + c_{n,4}}. \end{aligned}$$

Let $a_n^{\text {DRT}} (b_n, \psi )$ and $a_n^{\text {CSIT}} (b_n, \psi )$ represent the task allocation ratios of the Service Terminal, respectively. Similarly, let $a_{N+1}^{\text {DRT}} (b_{N+1}, \psi )$ and $a_{N+1}^{\text {CSIT}} (b_{N+1}, \psi )$ represent the task allocation ratios of the MEC server under two transmission mechanisms. The unique solution for $\psi _{N+1} (a_{N+1}, b_{N+1}) = \psi$ is given by the following equation:

33 $$\begin{aligned} \max \{a_{N+1}^{\text {DRT}} (b_{N+1}, \psi ), a_{N+1}^{\text {CSIT}} (b_{N+1}, \psi )\} \end{aligned}$$

Where

$$\begin{aligned} a_{N+1}^{\text {DRT}}(b_{N+1}, \psi )&= \frac{\psi }{\frac{c_{N+1,1}}{b_{N+1}} + c_{N+1,2} + c_{N+1,5}} \\ a_{N+1}^{\text {CSIT}}(b_{N+1}, \psi )&= \frac{\psi }{\frac{c_{N+1,3}}{b_{N+1}} + c_{N+1,4} + c_{N+1,5}} \end{aligned}$$

From 32 and 33, we can see that when $\psi > 0$ is fixed, the problem we are solving becomes maximizing the total task allocation ratio, introducing this problem as P2. Solving Problem P1, shows that, for any fixed value of $\psi$, there exists a feasible pair *a* and *b* whose objective value in Problem P1 is no greater than $\psi$. For problem P2, after the feasible relationship pair of problem P1 is determined, there exists a feasible solution whose objective value in problem P2 is at least 1. Based on the above analysis, when testing the feasibility of problem P1 for a given $\psi > 0$, solving problem P2 with the same $\psi$ is sufficient.

P2 $$\begin{aligned} \begin{aligned} \underset{b}{\max }\ F\left( b;\psi \right) = a_0\left( \psi \right) + \sum _{n=1}^{N+1} \max \left\{ a_{n}^{DRT}\left( b_n,\psi \right) , \right. a_{n}^{CSIT}\left( b_n,\psi \right) \Bigg \} \\ \text {s.t.} \quad 0 \leqslant \sum _{n=1}^{N+1} b_n \leqslant 1, \\ \quad 0 \leqslant b_n \leqslant 1, \, n=1,2,\dots ,N+1. \end{aligned} \end{aligned}$$

### IGWO over the OUV

The Grey Wolf Optimizer (TGWO) is a swarm-intelligence algorithm that models the leadership hierarchy and hunting behavior of grey wolves^32^. Compared with other swarm-intelligence algorithms, such as Particle Swarm Optimization (PSO) and the Firefly Algorithm, TGWO often converges to higher-quality near-optimal solutions. However, TGWO also has some drawbacks, such as a tendency to stagnate during the search phase and a gradually decreasing convergence speed in the later stages of the search. Accordingly, we adopt an IGWO to overcome these limitations and solve the joint optimization problem considered in this study.

#### Position update strategy

We denote the grey wolf population as $x_i, i = 0, 1, 2, \dots , m$, the fitness values of the grey wolves as $\psi _i, i = 0, 1, 2, \dots , m$, the position of the prey as $\psi ^*{(a,b)}$, and use the task allocation ratio *a* and bandwidth allocation ratio *b* as the position information for each grey wolf to update its fitness value $\psi$.

The position update for wolves encircling the prey is given by:

34 $$\begin{aligned} \begin{aligned} D_{\alpha }&= |c_1 \times X_{\alpha }^t - X^t| \\ D_{\beta }&= |c_2 \times X_{\beta }^t - X^t| \\ D_{\delta }&= |c_3 \times X_{\delta }^t - X^t| \end{aligned} \end{aligned}$$

35 $$\begin{aligned} {\left\{ \begin{array}{ll} X_1 = X_{\alpha } - A_1 \times D_{\alpha } \\ X_2 = X_{\beta } - A_2 \times D_{\beta } \\ X_3 = X_{\delta } - A_3 \times D_{\delta } \end{array}\right. } \end{aligned}$$

36 $$\begin{aligned} X(t+1) = \frac{X_1 + X_2 + X_3}{3} \end{aligned}$$

In this formulation, *t* denotes the iteration index. The vectors $X_\alpha ^t, X_\beta ^t, X_\delta ^t \text { and } X^t$ represent the positions of the prey and the grey wolves, respectively, while $\vec {A}$ and $\vec {C}$ are coefficient vectors, defined as follows:

37 $$\begin{aligned} {\left\{ \begin{array}{ll} \vec {A} = 2 \vec {m} \cdot \vec {r}_1 - \vec {m} \\ \vec {C} = 2 \cdot \vec {r}_2 \end{array}\right. } \end{aligned}$$

38 $$\begin{aligned} m = 2 - 2 \left( \frac{t}{T_{\max }} \right) \end{aligned}$$

In the formula, $T_{\max }$ represents the maximum iteration count. The parameter $m$ is a linearly decaying coefficient that varies from 2 down to 0; while $\vec {r_1}$ and $\vec {r_2}$ denote random variables taking values in the interval [0, 1].

#### CPM mapping initialization

We use CPM-based mapping to generate the initial population. CPM mapping combines piecewise mapping with Logistic mapping, allowing for the creation of chaotic sequence generators that possess both global chaotic characteristics and local features through piecewise control. This combined approach effectively enhances the diversity and uniformity of population initialization. The expression is as follows:

39 $$\begin{aligned} x_{n+1} {\left\{ \begin{array}{ll} \text {mod} \left( \left( \frac{x_n}{d} + \cos \left( n \cos ^{-1} (x_n \cdot \pi ) \right) \right) , 1 \right) , & 0 \le x_n< d \\ \text {mod} \left( \left( \frac{x_n - d}{0.5 - d} + \cos \left( n \cos ^{-1} (x_n \cdot \pi ) \right) \right) , 1 \right) , & d \le x_n< 0.5 \\ \text {mod} \left( \left( \frac{1 - d - x_n}{0.5 - d} + \cos \left( n \cos ^{-1} (x_n \cdot \pi ) \right) \right) , 1 \right) , & 0.5 \le x_n< 1 - d \\ \text {mod} \left( \left( \frac{1 - x_n}{d} + \cos \left( n \cos ^{-1} (x_n \cdot \pi ) \right) \right) , 1 \right) , & 1 - d \le x_n < 1 \end{array}\right. } \end{aligned}$$

Where $d$ is a control parameter with a value range of (0, 1).

#### Adaptive grouping strategy

During the search for the optimal solution, we design an adaptive grouping mechanism to partition the wolf population. Specifically, wolves are divided into three groups according to their distance from the prey, which serves as an indicator of their fitness. These groups are the hunting group $X_{i,a}$, the wandering group $X_{i,b}$ and the searching group $X_{i,c}$. The expression is as follows:

40 $$\begin{aligned} X_{i,j} = {\left\{ \begin{array}{ll} X_{i,a}, & 1 \le a< m_1 \\ X_{i,b}, & m_1 \le a< m_2 \\ X_{i,c}, & m_2 \le a < m \end{array}\right. } \end{aligned}$$

$m_1$ and $m_2$ are the boundaries that define the groups. Specifically, the wolves are ranked by fitness: the highest-fitness individuals are assigned to the predator group, those with intermediate performance belong to the wanderer group, and the lowest-fitness wolves are placed in the searcher group. The expressions for $m_1$ and $m_2$ are as follows:

41 $$\begin{aligned} {\left\{ \begin{array}{ll} m_1 = \frac{1}{4} N \left( 1 - \frac{t}{T_{\max }} \right) ^{\frac{1}{2}} \\ m_2 = N - \frac{1}{4} N \left( \frac{t}{T_{\max }} \right) ^{\frac{1}{2}} \end{array}\right. } \end{aligned}$$

As the iterations progress, the wolf-pack group boundaries are adaptively updated. At the initial iterations, the wolves are widely scattered, so a relatively rapid convergence is required. Therefore, a larger number of individuals are assigned to the predator group at this stage to enable quicker convergence to the current optimal solution. As the iteration proceeds and the wolf pack tends to converge, the need to escape local optima becomes more critical. Consequently, the searcher group, which is better suited for global exploration, increases in size as the iteration progresses.

#### Grouping position update strategies

The predator group updates its positions via an improved differential evolution strategy, enhancing local search and accelerating convergence. The update rule is as follows:

42 $$\begin{aligned} X_i^{t+1} = X_a^t + W \times \left( \frac{X_\beta ^t}{2} + \frac{X_\delta ^t}{2} - X_i^t \right) \end{aligned}$$

For the wanderer group, we employ a random reverse learning strategy for position updates. The specific update formula is as follows:

43 $$\begin{aligned} x_{i,j}' = UB_j + LB_j - r_3 \cdot x_{ij} \end{aligned}$$

$r_3$ is a random factor in the range [0, 1]. Compared with conventional opposition-based learning, the random reverse learning strategy generates dynamic candidate solutions, thereby increasing diversity and strengthening the algorithm’s search capability. In the position iterations of the wanderer group, we retain the better individuals from both the TGWO position update strategy and the random reverse learning strategy.

The search group employs position updates using multiplication and division operators from the Arithmetic Optimization Algorithm (AOA). The AOA algorithm is a meta-heuristic algorithm that updates individual positions based on arithmetic operators (addition, subtraction, multiplication, and division), offering good local and global search capabilities [31]. The specific update formula is as follows:

44 $$\begin{aligned} x_{i,j}^t = {\left\{ \begin{array}{ll} x_{\alpha ,j} \times \text {MOP} \times \left( (UB_j - LB_j) \times \mu - LB_j \right) , & r_4 < 0.5 \\ \frac{x_{\alpha ,j}}{\text {MOP} + \epsilon } \times \left( (UB_j - LB_j) \times \mu - LB_j \right) , & \text {otherwise} \end{array}\right. } \end{aligned}$$

Here, $\varepsilon$ is a non-zero decimal number, The term $x_{i,j}^{t+1}$ represents the j-th component of the i-th candidate solution at iteration $t+1$; whereas $x_{\alpha ,j}$ denotes the j-th component of the current wolf $\alpha$. The symbols $UB_j$ and $LB_j$ specify the lower and upper bounds of the j-th component, respectively. The variable $r_4$ is a random number drawn from the interval [0, 1]. MOP is a control parameter that adjusts the search process, and when $\mu = 0.5$ , the AOA algorithm achieves optimal performance. The probability function MOP is calculated as follows:

45 $$\begin{aligned} \text {MOP}(t) = 1 - \frac{\frac{1}{t^{\lambda }}}{\frac{1}{T_{\max }^{\lambda }}} \end{aligned}$$

Here, $\lambda$ is the sensitivity coefficient, and the AOA algorithm achieves optimal performance when $\lambda$ is equal to 5.

### Feasibility check

Using IGWO to solve problem P1, we can obtain the value of $\psi > 0$ and the allocation ratio combination of a and b from the position information of the $\alpha$ wolf in the algorithm’s output. To determine whether one can find a feasible pair $a$ and $b$ for which the objective $\psi (a, b)$ in problem P1 does not exceed a given constant $\psi > 0$.

Next, we show how the improved GPM algorithm can be applied to Problem P2. When using the traditional GPM algorithm to address problem P2, it necessitates calculating the objective function’s gradient, taking a step in the direction of the gradient, and then projecting the solution at that point onto the feasible set. However, because the objective of P2 contains a max operator, the objective function becomes non-smooth, making gradient calculation very challenging. However, because the objective function of P2 contains a max operator, rather than directly maximizing the objective function itself.

During the process of solving problem P2 with respect to $b$ , we assume the current point of $b$ is $b_t$ , where represents the iteration count. With the present bandwidth allocation for $b_t$ , we can determine the choice between the DRT and CSIT transmission strategies. Let $N_t^{DRT}$ denote the set of SNs selecting the DRT strategy and $N_t^{CSIT}$ the set selecting CSIT. The GPM algorithm is utilized to address Problem P3, and the returned solution for $b_t$ can be divided into two cases: Case 1, the transmission sets $\mathscr {N} _t^{DRT}$ and $\mathscr {N} _t^{CSIT}$ induced by the updated $b_{t+1}$ differ from the current strategy; and Case 2, where the transmission strategies $\mathscr {N} _t^{DRT}$and $\mathscr {N} _t^{DRT}$ $\mathscr {N} _t^{CSIT}$ based on $b_{t+1}$ are the same as the current strategy. When Case 1 occurs, we have the following relational expression:

46 $$\begin{aligned} F(b_{t+1}; \psi ) \ge F_t(b_{t+1}; \psi ) \ge F_t(b_t; \psi ) = F(b_t; \psi ) \end{aligned}$$

The above equation implies that the objective value of Problem P2 increases monotonically across iterations and, after a finite number of updates, converges to the upper bound of Problem P2. The first inequality follows from the fact that $F_t(b; \psi )$ provides a lower bound for $F(b; \psi )$ . The second inequality is ensured because, when Problem P3, is handled by the GPM algorithm, each iteration performs a gradient–ascent update and therefore yields a larger objective value for Problem P2. The equality is an immediate consequence of the definition of $F_t(b; \psi )$. In Case 2, the point generated by applying GPM to Problem P3 is stationary; since the corresponding transmission strategy coincides with that of Problem P2, this point is also a stationary point of ProblemP2.

Finally, since the objective function of Problem P3 is smoother than that of Problem P2, solving problem P3 using the GPM algorithm becomes feasible. For the SNs, the gradient calculation is given by the following formula:

P3 $$\begin{aligned} \begin{aligned} \underset{b}{\max }\ F\left( b; \psi \right) = a_0\left( \psi \right) + \sum _{n \in N_t^{\text {DRT}}} a_n^{\text {DRT}}\left( b_n, \psi \right) + \sum _{n \in N_t^{\text {CSIT}}} a_n^{\text {CSIT}}\left( b_n, \psi \right) \\ \text {s.t.} \quad 0 \leqslant \sum _{n=1}^{N+1} b_n \leqslant 1, \\ \quad 0 \leqslant b_n \leqslant 1, \, n=1,2,\dots ,N+1. \end{aligned} \end{aligned}$$

47 $$\begin{aligned} \frac{\partial F_t(b; \psi )}{\partial b_n} = {\left\{ \begin{array}{ll} \frac{\psi c_{n,1}}{\left( c_{n,1} + c_{n,4}b_n\right) ^2}, & \text {if } V_n \in \mathscr {N}_t^{\text {DRT}} \\ \frac{c_{n,2}}{b_n^2\left( c_{n,3} + c_{n,4}\right) }, & \text {if } V_n \in \mathscr {N}_t^{\text {CSIT}} \end{array}\right. } \end{aligned}$$

48 $$\begin{aligned} \frac{\partial F_t(b; \psi )}{\partial b_{N+1}} = {\left\{ \begin{array}{ll} \frac{\psi c_{N+1,1}}{\left( c_{N+1,1} + b_{N+1}c_{N+1,2} + b_{N+1}c_{N+1,5}\right) ^2}, & \text {if } V_{N+1} \in \mathscr {N}_t^{\text {DRT}} \\ \frac{\psi c_{N+1,3}}{\left( c_{N+1,3} + b_{N+1}c_{N+1,4} + b_{N+1}c_{N+1,5}\right) ^2}, & \text {if } V_{N+1} \in \mathscr {N}_t^{\text {CSIT}} \end{array}\right. } \end{aligned}$$

**Complexity and Computational Overhead Analysis**

To justify the practical applicability of the proposed IGWO-FCA, we analyze its computational complexity, particularly in comparison with Deep Reinforcement Learning (DRL)-based methods. Let $N_{\text {pop}}$, $T_{\text {max}}$, *D*, and *K* denote the wolf population size, the maximum number of iterations, the dimension of the decision variables, and the number of projection steps in the Feasibility Checking Algorithm (FCA), respectively.Since the fitness evaluation and position update of each individual are both bounded by *O*(*D*), the computational complexity per IGWO iteration is $O(N_{\text {pop}} D)$. Therefore, the overall complexity of the IGWO phase is $O(N_{\text {pop}} T_{\text {max}} D)$. In addition, the FCA based on the Gradient Projection Method has complexity *O*(*KD*). Accordingly, the overall complexity of the proposed method can be expressed as $O(N_{\text {pop}}T_{\text {max}} D+KD)$. This complexity grows linearly with the population size, iteration budget, and decision dimension, which makes the computational overhead predictable and acceptable for the central control unit. In contrast, although DRL-based methods can achieve fast inference after training, their training stage usually requires extensive environment interaction and repeated parameter updates for deep neural networks. Therefore, compared with training-based approaches, the proposed IGWO-FCA does not require an offline training phase and can be executed directly for each offloading decision, which makes it more suitable for real-time CCTS optimization in smart mining scenarios.

## Results and discussion

**Fig. 3:**
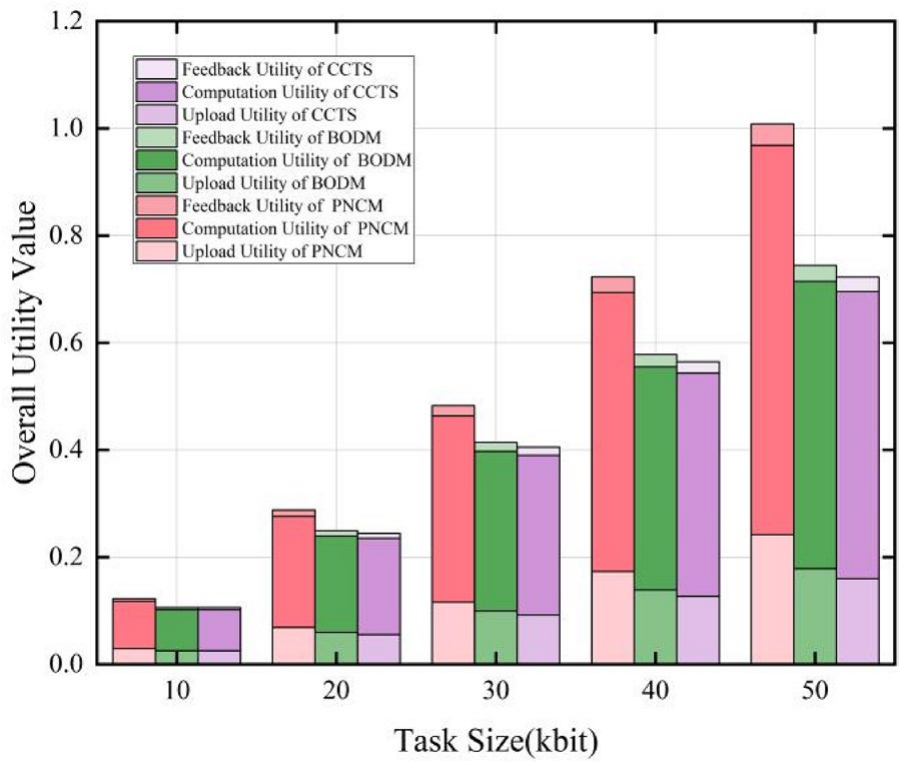
Comparison of system utility values versus $S_{data}$ for different mechanisms.

**Fig. 4:**
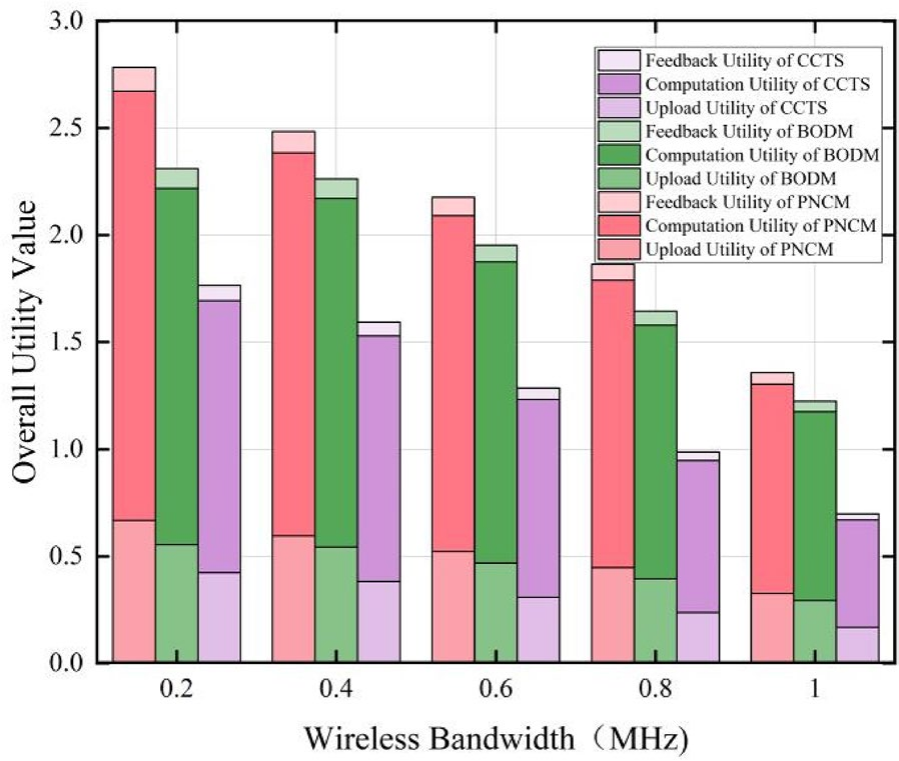
The relationship between $B_{total}$ and system utility values under different mechanisms.

**Fig. 5:**
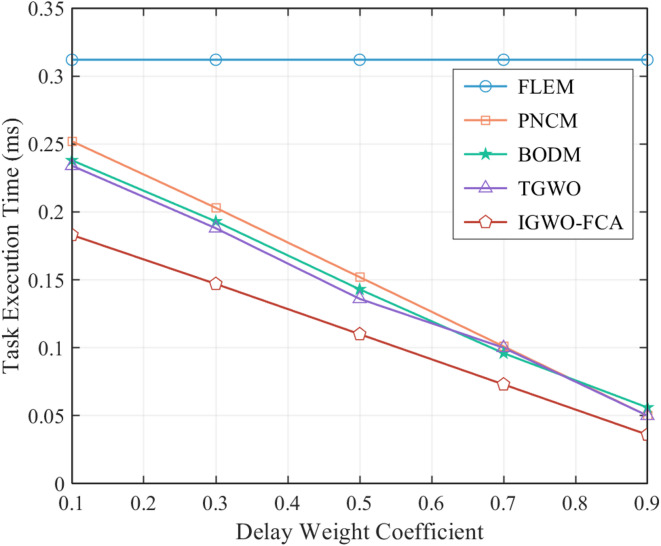
The impact of the delay weight coefficient $\mu$ on the system’s total delay.

**Fig. 6:**
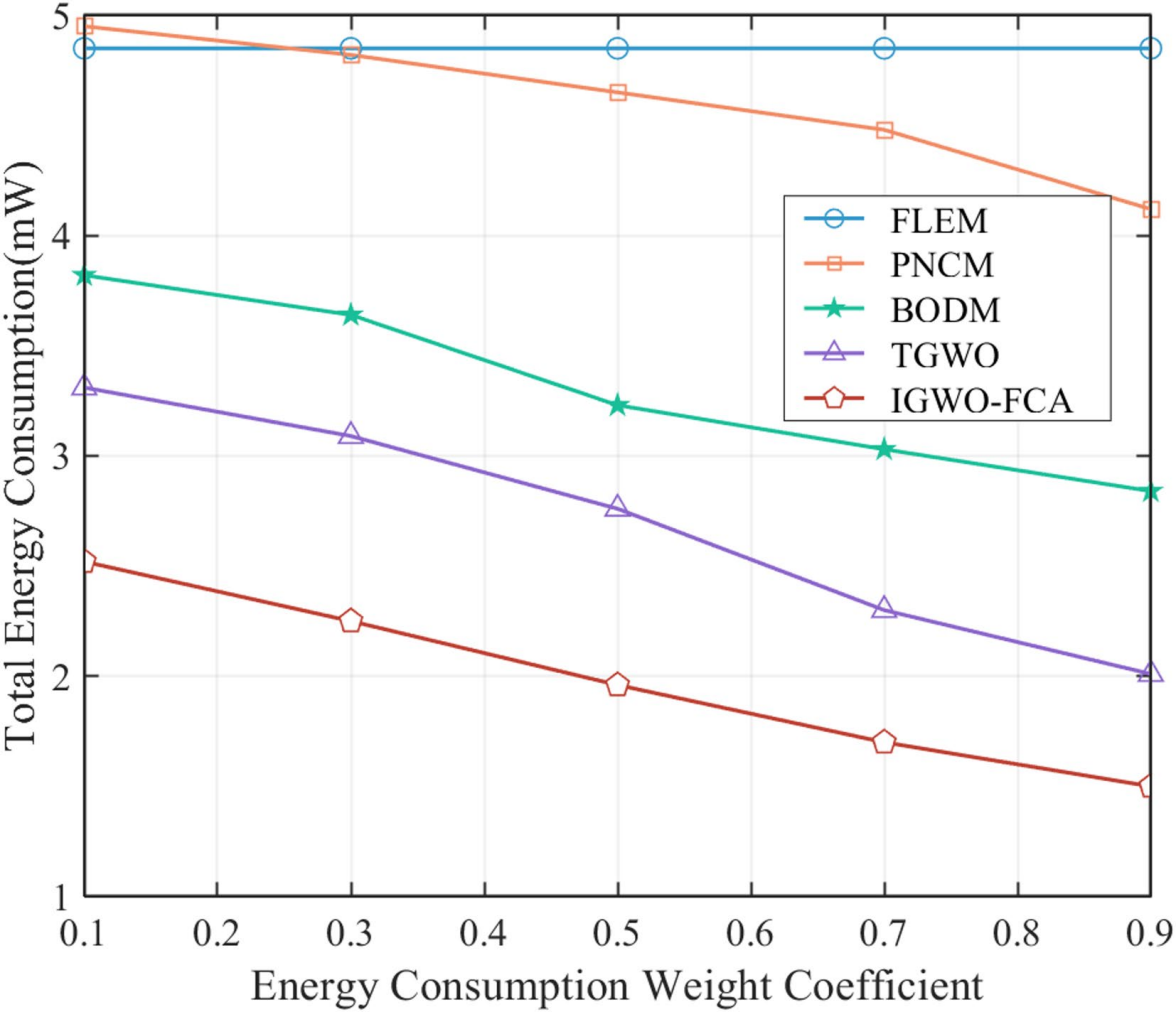
The impact of the energy consumption weight coefficient $\nu$ on the system’s total energy.

**Fig. 7:**
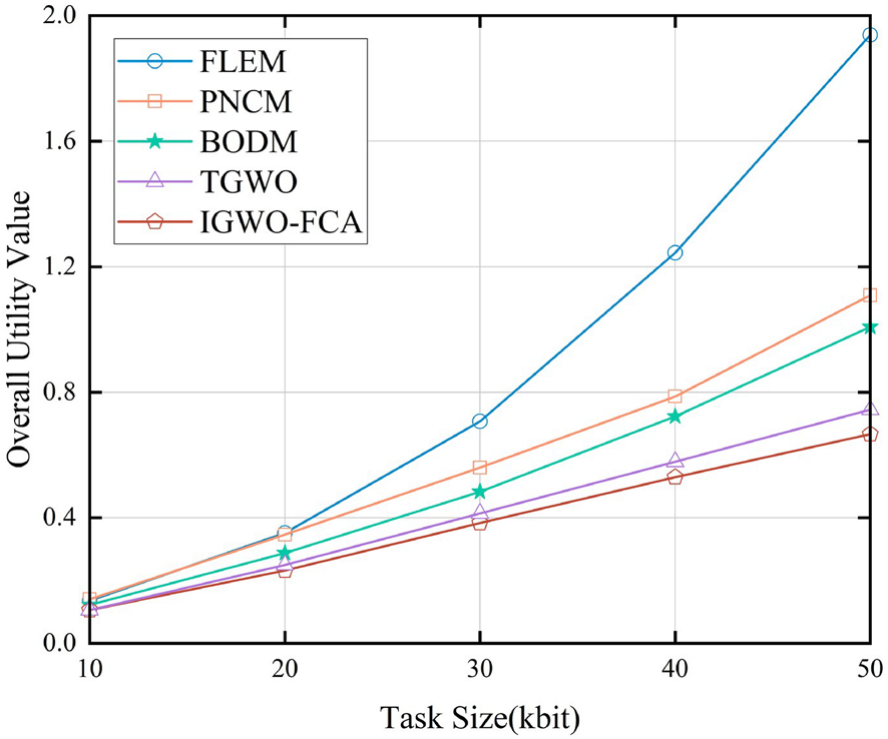
Performance Comparison for Different $S_{data}$.

**Fig. 8:**
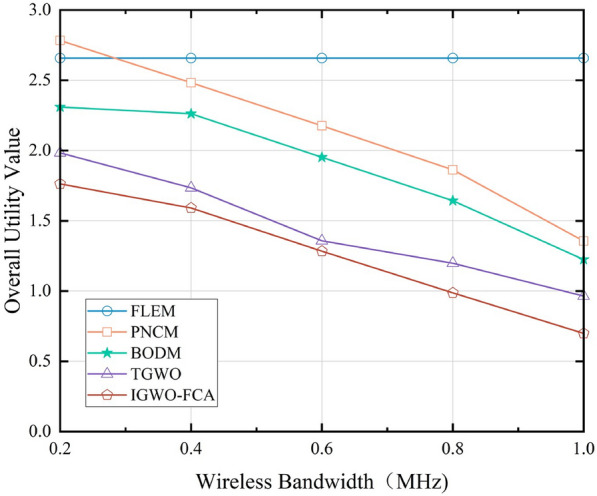
Performance Comparison for Different $B_{total}$.

**Fig. 9:**
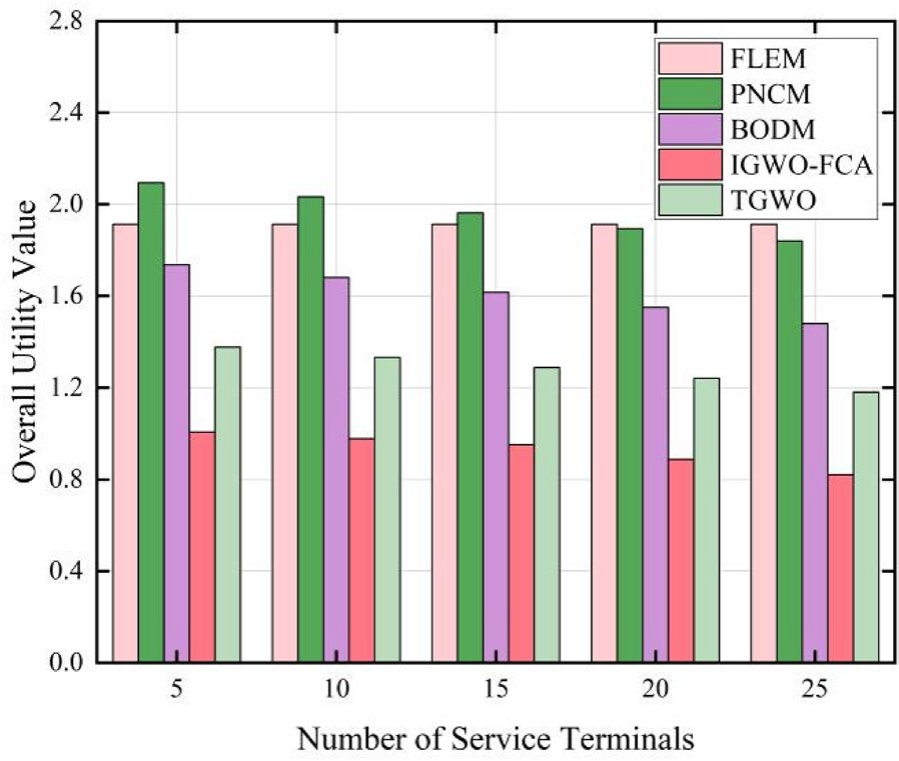
Performance Comparison of Different Numbers of Terminals.

In this section, we conducted experiments on the MATLAB 2020b simulation platform to validate the proposed mechanisms. A mining task offloading model was constructed, including task terminals (TaT), service terminals (ST), and a base station hosting an edge server. The effectiveness of the proposed CCTS and the computational efficiency of the IGWO-FCA algorithm were validated. We assumed that a service base station (BS) with a coverage area of 300 meters is deployed at a mining face, and within its coverage area, there is only one TaT and up to 25 service terminals randomly distributed. In the simulations, all nodes are assumed to remain static during one offloading decision period. Therefore, mobility is not explicitly modeled, and the channel gain is determined by the spatial placement in each simulation realization. Unless otherwise stated, each reported data point in Figs. 3, 4, 5, 6, 7, 8 and 9 was obtained by averaging the results over multiple independent simulation runs under the same parameter setting. The key parameter information for the system simulation is shown in Table 1.

**Table 1:** System parameters used in original paper figures.

System parameters	
Parameters	Values
$B_{\text {total}}$	$0.21\,\text {MHz}$
$S_{\text {data}}$	10–$50\,\text {kb}$
$f_0$	$1.0 \times 10^{9}\,\text {cycles/s}$
$f_n$	$2.0 \times 10^{9}\,\text {cycles/s}$
$f_{N+1}$	$8.0 \times 10^{9}\,\text {cycles/s}$
$p_0/p_n$	0.5–$1.0\,\text {W}$
$p_{N+1}$	$2.0\,\text {W}$
$\sigma _n, \sigma _{N+1}$	$1 \times 10^{-9}\,\text {W}$
$R_{wired}$	$10\,\text {Gbps}$
*k*	$1 \times 10^{-28}$
*M*	$1000\,\text {cycles/bit}$

We benchmark the proposed IGWO-FCA against the following four task-offloading schemes:

- TGWO (Traditional Grey Wolf Optimization): Utilizes the traditional Grey Wolf Optimization algorithm to solve the optimization problem mentioned in the paper^33^.
- BODM (Binary Offloading Decision Mechanism): Tasks can be executed on the MEC server, on service terminals, or locally, but the offloading ratio can only be 0 or 1^34^.
- PNCM (Partially Noncooperative Mechanism): Tasks may be executed locally or offloaded to the MEC server, but not to the service terminal^35^.
- FLEM (Fully Local Execution Mechanism): All computational tasks are executed locally^36^.

### Effectiveness of CCTS

In this section, we propose two benchmark mechanisms to compare with the CCTS. By adjusting the parameters of the task and bandwidth size, we focus on the differences in upload utility values among the three, thereby verifying the effectiveness of our proposed CCTS. The first benchmark, PNCM, involves only the MEC server and the TaT in the offloading process, with tasks executed locally or offloaded to the MEC server. The second benchmark mechanism, BODM, allows the participation of the ST but does not consider task partitioning or bandwidth resource allocation. The task offloading ratio can only be 0 or 1.

Figure 3 shows the effect of the input data size $S_{data}$ on the system utility of CCTS, BODM, and PNCM. As $S_{data}$ increases, the system utility values for all three mechanisms grow linearly. Furthermore, the performance of CCTS is consistently better than that of BODM and PNCM. When $S_{data}$ is less than 20 kbit, the system utility values of CCTS and BODM are similar and lower than that of PNCM. When $S_{data}$ increases to 30 kbit, the system utility values of both BODM and PNCM exceed those of CCTS. As $S_{data}$ continues to increase, the gap between the three mechanisms gradually widens. Since our proposed CCTS reduces the system utility value by optimizing the selection of the upload strategy, we focus more on the utility value of the upload process. Compared to the BODM and PNCM benchmark mechanisms, the average upload utility value is reduced by 6.3% and 22.9%, respectively.

Figure 4 shows the effect of the total wireless bandwidth $B_{total}$ on the system utility. As $B_{total}$ increases, the system utility values for all three mechanisms decrease linearly. Moreover, the performance of CCTS consistently surpasses that of BODM and PNCM. Since $B_{total}$ directly affects the uplink transmission rate, the curves for all three mechanisms exhibit more pronounced changes. Within the CCTS, each service terminal first utilizes its locally sensed data to alleviate the stringent constraint associated with $B_{total}$. With a larger $B_{total}$, the system incrementally assigns input-data transmission to more service terminals, which further enlarges the performance gap between CCTS and the other two baselines. This assessment concludes that CCTS consistently outperforms BODM and PNCM. Importantly, when wireless bandwidth constrains the system, CCTS achieves a markedly larger improvement than the conventional PNCM scheme. Compared to the benchmark mechanisms BODM and PNCM, the average upload utility value is reduced by 41.8% and 34.1%, respectively.

### The effect of the weight coefficient

Figures 5 and 6 analyze the influence of the weighting coefficients $\mu$ and $\nu$ on total delay and energy consumption. As the delay weight coefficient increases from 0.1 to 0.9, the PNCM, BODM, TGWO, and IGWO-FCA algorithms all exhibit reductions in total delay and energy consumption. However, FLEM, due to its local execution strategy, is unaffected by the weighting coefficients, and its total delay and energy consumption remain constant. For every weighting coefficient, IGWO-FCA yields the minimum total delay and energy consumption, outperforming TGWO, BODM, PNCM, and FLEM. The average total delay of IGWO-FCA decreases by 25.1%, 37.5%, 40.3%, and 60.2%, respectively, while the total energy consumption decreases by 34.8%, 48.4%, 49.5%, and 61.7%.

### Performance comparison of different algorithms

Figure 7 presents the effect of different input data sizes on the delay, energy consumption, and utility of different algorithms. As the input data size increases, the delay, energy consumption, and system utility values generally rise. The proposed IGWO-FCA consistently outperforms the other four offloading strategies. However, when the input data size exceeds 30 kbit, the system utility values of the BODM, PNCM, and FLEM algorithms increase significantly. This is because the constrained computing capabilities of both the MEC server and the TaT limit their ability to satisfy the heavy computational requirements of latency-sensitive and computation-intensive tasks. By adopting the proposed CCTS and IGWO-FCA, we can effectively address the issue of resource constraints. Compared to the TGWO, BODM, PNCM, and FLEM schemes, the IGWO-FCA achieves an average reduction in system utility value of 10.3%, 12.7%, 14.8%, and 17.9%, respectively.

Figure 8 examines how the available bandwidth affects the delay, energy consumption, and utility of different algorithms. As bandwidth resources increase, the delay, energy consumption, and system utility values gradually decrease for all algorithms except FLEM. Since tasks in FLEM are executed locally and are not affected by wireless bandwidth, its performance remains constant. In contrast, the proposed IGWO-FCA consistently achieves the lowest delay, energy consumption, and system utility across all scenarios. Compared with the TGWO, BODM, PNCM, and FLEM schemes, the IGWO-FCA achieves an average reduction in system utility value of 13.9%, 34.1%, 41.8%, and 52.7%, respectively.

Figure 9 evaluates the scalability of different algorithms with respect to the number of service terminals. As shown, the utility values decrease to varying extents as the number of service terminals increases. Among the five schemes, FLEM and PNCM perform poorly, indicating that local computation and noncooperative offloading modes cannot effectively support users’ computational tasks. In contrast, IGWO-FCA, BODM, and TGWO effectively reduce the system utility values through cooperative offloading modes. Overall, our proposed IGWO-FCA achieves the lowest system utility in all cases. Compared to the TGWO, BODM, PNCM, and FLEM schemes, the IGWO-FCA reduces the system utility by an average of 27.6%, 42.3%, 52.7%, and 51.0%. Within the tested range of up to 25 service terminals, no clear saturation point is observed. The exact saturation threshold under denser deployments will be investigated in future work.

## Conclusions

This paper investigates task offloading and resource allocation in edge computing for collaborative communication and sensing in mining environments. We introduce a novel CCTS that dynamically selects the transmission mode for both input-data uploads and computation instructions, adapting to real-time network conditions. By jointly optimizing the task transmission method, task allocation ratio, and bandwidth allocation ratio, we effectively minimize the system-level SL-SEC. The proposed IGWO-FCA efficiently solves this optimization problem, producing high-quality solutions. Simulation results demonstrate that CCTS consistently outperforms benchmark schemes across multiple performance metrics. In this study, we focus specifically on optimizing task and bandwidth allocation, as well as adaptively selecting transmission strategies to fully leverage system resources. All baseline schemes are implemented under identical system models and constraints, without utilizing sensing-assisted transmission, ensuring fair and meaningful comparisons. Although the central control unit is assumed to have periodic access to network and computing-state information, practical scenarios may involve delays or noisy data. Despite such uncertainties, the core CCTS decision (DRT vs. CSIT) remains robust under typical conditions. Nonetheless, dependencies among tasks after partitioning represent an important research avenue. In future work, we plan to explicitly incorporate subtask dependencies and investigate robust optimization or predictive learning approaches to mitigate the performance degradation caused by imperfect and delayed information in dynamic mine environments.

## Data Availability

The datasets generated during and/or analysed during the current study are available from the corresponding author on reasonable request.
